# COVID-19 infection risk amongst 14,104 vaccinated care home residents: a national observational longitudinal cohort study in Wales, UK, December 2020–March 2021

**DOI:** 10.1093/ageing/afab223

**Published:** 2021-11-17

**Authors:** Joe Hollinghurst, Laura North, Malorie Perry, Ashley Akbari, Mike B Gravenor, Ronan A Lyons, Richard Fry

**Affiliations:** Population Data Science and Health Data Research UK, Swansea University, Swansea, Wales SA2 8PP, UK; Population Data Science and Health Data Research UK, Swansea University, Swansea, Wales SA2 8PP, UK; Vaccine Preventable Disease Programme and Communicable Disease Surveillance Centre, Public Health Wales, 2 Capital Quarter, Tyndall Street, Cardiff CF10 4BZ, Wales, UK; Population Data Science and Health Data Research UK, Swansea University, Swansea, Wales SA2 8PP, UK; Health Data Science, Swansea University Medical School, Swansea University, Swansea,Wales SA2 8PP, UK; Population Data Science and Health Data Research UK, Swansea University, Swansea, Wales SA2 8PP, UK; Population Data Science and Health Data Research UK, Swansea University, Swansea, Wales SA2 8PP, UK

**Keywords:** COVID-19, SARS-CoV-2, vaccination, care homes, older people

## Abstract

**Background:**

vaccinations for COVID-19 have been prioritised for older people living in care homes. However, vaccination trials included limited numbers of older people.

**Aim:**

we aimed to study infection rates of SARS-CoV-2 for older care home residents following vaccination and identify factors associated with increased risk of infection.

**Study Design and Setting:**

we conducted an observational data-linkage study including 14,104 vaccinated older care home residents in Wales (UK) using anonymised electronic health records and administrative data.

**Methods:**

we used Cox proportional hazards models to estimate hazard ratios (HRs) for the risk of testing positive for SARS-CoV-2 infection following vaccination, after landmark times of either 7 or 21 days post-vaccination. We adjusted HRs for age, sex, frailty, prior SARS-CoV-2 infections and vaccination type.

**Results:**

we observed a small proportion of care home residents with positive polymerase chain reaction (tests following vaccination 1.05% (N = 148), with 90% of infections occurring within 28 days. For the 7-day landmark analysis we found a reduced risk of SARS-CoV-2 infection for vaccinated individuals who had a previous infection; HR (95% confidence interval) 0.54 (0.30, 0.95). For the 21-day landmark analysis, we observed high HRs for individuals with low and intermediate frailty compared with those without; 4.59 (1.23, 17.12) and 4.85 (1.68, 14.04), respectively.

**Conclusions:**

increased risk of infection after 21 days was associated with frailty. We found most infections occurred within 28 days of vaccination, suggesting extra precautions to reduce transmission risk should be taken in this time frame.

## Key Points

- Increased risk of infection after 21 days was associated with frailty.
- Most (90%) positive PCR tests occurred within 28 days of vaccination.
- Extra precautions to reduce transmission risk should be taken within 28 days of vaccination.

## Introduction

Vaccinations for SARS-CoV-2 in the UK have been prioritised to older people living in care homes [1, 2]. However, the efficacy of the COVID-19 vaccine in older people is relatively unknown, with very few trials recruiting older people and older people with frailty [3]. Specifically, the Oxford–AstraZeneca vaccine trials had less than 4% of participants over 70 years of age and those with comorbidities were a minority [4]. Similarly, the initial Pfizer BioNTECH trials included only 774 individuals aged 75 or over and 3,848 individuals aged 65 and over from a total of 18,198 vaccinated individuals [5].

Care homes are a keystone of adult social care. They provide accommodation and care for those needing substantial help with personal care, but more than that, they are people’s homes [6, 7]. In 2016, there were 11,300 care homes in the UK, with a total of 410,000 residents [8]. Within care homes, people live in proximity and may live with frailty and many different health conditions, making them susceptible to outbreaks of infectious disease [6]. COVID-19 is described by Lithande *et al.* [9], as ‘…a dynamic, specific and real threat to the health and well-being of older people’ (2020, p. 10). The impacts of COVID-19 on this sub-population have been reported widely in both international and UK media, and in a growing peer reviewed literature.

Here, we produced a rapid report, in near real-time, on the risk of SARS-CoV-2 infection for vaccinated care home residents. This is the first study we are aware of investigating this vulnerable sub-population. Furthermore, we included information on previous positive SARS-CoV-2 polymerase chain reaction (PCR) tests, age, sex and frailty. We were able to do this using the existing infrastructure and linked data from the Secure Anonymised Information Linkage (SAIL) Databank [10–12].

## Methods

### Study design and setting

We conducted an observational data-linkage study for older care home residents in Wales (UK). We used data on 14,104 individuals receiving a SARS-CoV-2 vaccination from 4 December 2020 to 12 February 2021 and testing data from 4 December 2020 to 4 March 2021 to investigate positive SARS-CoV-2 PCR tests following a vaccination.

### Data sources

We used linked longitudinal data from the SAIL Databank to create our datasets [10–12]. Specifically, we used the COVID Vaccine Dataset (CVD) to identify individuals living in care homes who had received a vaccination. We included all individuals identified as an ‘older adult resident in a care home’. The Pathology COVID-19 Daily data was used to identify dates of positive SARS-CoV-2 PCR tests. A cleaned and pre-linked version of the Welsh Demographic Service Dataset was used to determine demographic information for each individual [13]. We also linked to the Patient Episode Database for Wales (PEDW) to include an indication of frailty.

### Hospital frailty risk score

The Hospital Frailty Risk Score (HFRS) was developed using Hospital Episode Statistics (HES), a database containing details of all admissions, emergency department attendances and outpatient appointments at NHS hospitals in England, and validated on over one million older people using hospitals in 2014/15 [14]. The HFRS uses the International Classification of Disease version 10 (ICD-10) [15] codes to search for specific conditions from secondary care. A weight is then applied to the conditions and a cumulative sum is used to determine a frailty status of low, intermediate or high. We additionally included a HFRS score of ‘No score’ for people who had not been admitted to hospital in the look back period. We calculated the HFRS using the PEDW, the Welsh counterpart to HES, on the vaccination date, with a two year look back of all hospital admissions recorded in Wales.

### Variables

Our outcome of interest was the time to a positive SARS-CoV-2 PCR test following a vaccination. Individuals were censored for death or the end of study period. We included covariates for previous positive SARS-CoV-2 PCR tests (yes/no), age (continuous), sex (male/female), frailty (HFRS: no score, low, intermediate, high), and vaccine manufacturer (Oxford–AstraZeneca, Pfizer-BioNTECH). Previous positive SARS-CoV-2 PCR tests were identified at any time point before vaccination.

**Table 1:** Demographic information for the total cohort and the cohort stratified by those who had a subsequent positive SARS-CoV-2 PCR test following vaccination

		Positive PCR test post-vaccine	
	Total	No	Yes
N	14,104	13,956 (98.95%)	148 (1.05%)
Previous COVID infection	2,868 (20.3%)	2,848 (20.4%)	20 (13.5%)
Mean age (SD)	85 (8.3)	85.1 (8.3)	84.5 (8.4)
Sex			
Male	4,096 (29.0%)	4,045 (29.0%)	51 (34.5%)
Female	10,008 (71.0%)	9,911 (71.0%)	97 (65.5%)
HFRS			
No Score	6,409 (45.4%)	6,356 (45.5%)	53 (35.8%)
Low	1,149 (8.1%)	1,138 (8.2%)	11 (7.4%)
Intermediate	3,009 (21.3%)	2,969 (21.3%)	40 (27%)
High	3,537 (25.1%)	3,493 (25.0%)	44 (29.7%)
Vaccine Type			
Oxford–Astrazeneca	12,571 (89.1%)	12,459 (89.3%)	112 (75.7%)
Pfizer-BioNTECH	1,533 (10.9%)	1,497 (10.7%)	36 (24.3%)

SD, Standard deviation

**Figure 1:**
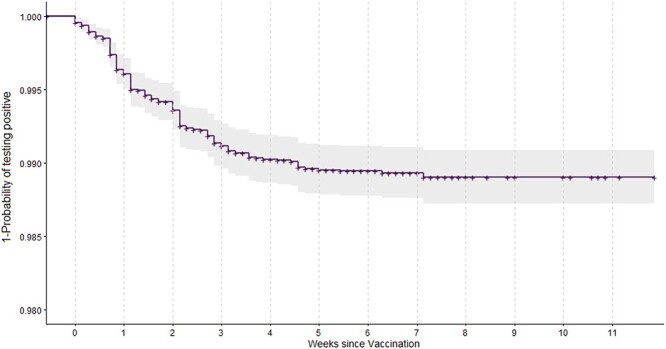
Kaplan–Meier curve for the time to the first positive SARS-COV-2 PCR test following vaccination.

### Statistical methods

We included basic demographic information and investigated differences between individuals who had a positive SARS-CoV-2 PCR test following vaccination. We produced a Kaplan–Meier survival curve and an empirical cumulative distribution function (CDF) for the time to first positive SARS-CoV-2 PCR test following vaccination. We also calculated hazard ratios (HR) for our covariates using univariable and multivariable Cox proportional hazards models. For the Cox proportional hazards models, we defined two landmark periods for immunisation, 7 and 21 days. In a landmark analysis, only those who have not had an event (positive PCR test) for the specified time period are included. In other words, individuals who had a positive SARS-CoV-2 PCR test within the 7 and 21-day periods were removed from the respective analyses, see Figure 3 for an example. We included these periods as a proxy for the varying number of days for the vaccine to become effective. As a sensitivity analysis, we repeated the analysis with the 7 and 21-day immunisation (landmark) periods, but applied a maximum follow-up of 14 days. Individuals were right censored for death or the end of the study period, whichever occurred first. Violations of the proportional hazards assumption were tested for using Schoenfeld residuals.

## Results

**Figure 2:**
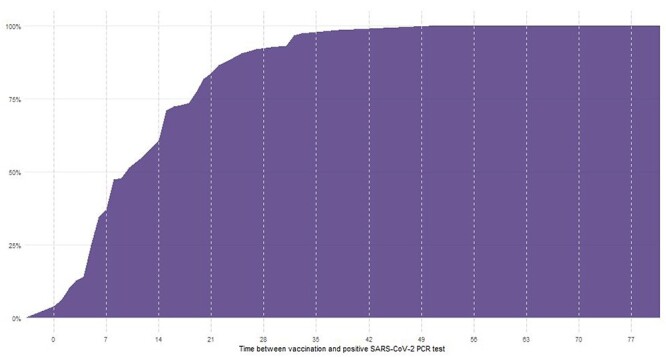
CDF for the time between vaccination and first positive PCR test (N = 148).

We identified 14,501 vaccinated older adult residents in a care home in the CVD dataset. We removed 240 residents prior to analysis because of incomplete demographic information. We restricted the age group to those aged 60+, removing a further 157 individuals, resulting in 14,104 residents used for analysis. The basic demographic information for the total cohort and the cohort stratified by those who had a subsequent positive SARS-CoV-2 PCR test following vaccine is presented in Table 1. Figure 1 shows the Kaplan–Meier curve for the time to first positive PCR test following vaccination. The curve indicates an overall small proportion of individuals testing positive following vaccination. Figure 2 shows the empirical CDF for the times between first positive PCR test and vaccination. The Kaplan–Meier curve and empirical CDF suggest a susceptible period of vaccinated individuals up to 42 days, with approximately 40% of individuals having a positive PCR test within 7 days, 60% within 14 days, 85% within 21 days, 90% within 28 days and over 95% within 35 days.

**Figure 3:**
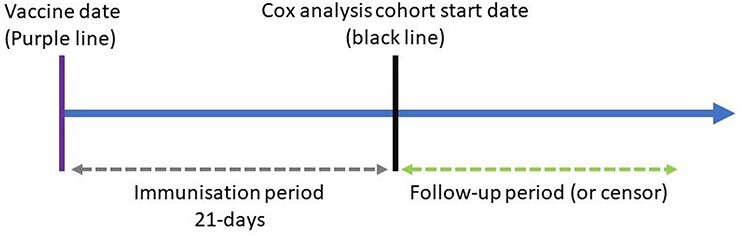
Immunisation (landmark) analysis timeline example.

HRs and 95% confidence intervals (CIs) for the Cox proportional hazards models are presented in Tables 2 and 3. In our multivariable analyses, we found a reduced risk of SARS-CoV-2 infection for vaccinated individuals who had a previous infection after a 7-day immunisation period; HR (95% CI) 0.54 (0.30, 0.95), and an increased risk for those receiving the Pfizer-BioNTECH vaccine HR 3.83 (2.45, 5.98). The 21-day immunisation period multivariable model indicated frailty as a risk factor, with low frailty having a HR of 4.59 (1.23, 17.12) and intermediate frailty with a HR of 4.85 (1.68, 14.04). The Schoenfeld residual test indicated only the 21-day immunisation/14-day observation model deviated from the proportional hazards assumption at the 95% level (*P* value 0.04), all other models met proportional hazards assumptions.

**Table 2:** Univariable and multivariable Cox proportional hazards models with 7 and 21-day landmark times applied to the cohort. HRs and presented with 95% CIs. Results that are statistically significant at the 95% level are highlighted in bold font

	7-day landmark time		21-day landmark time	
HRs (95% CI)	Univariable	Multivariable	Univariable	Multivariable
Age	1.004 (0.98, 1.028)	1.01 (0.985, 1.036)	1.024 (0.976, 1.074)	1.031 (0.981, 1.084)
Previous positive PCR test (reference: no)				
Yes	0.667 (0.379, 1.175)	**0.535 (0.301, 0.949)**	0.931 (0.353, 2.46)	0.807 (0.301, 2.159)
Sex (reference: female)				
Male	1.39 (0.919, 2.104)	1.428 (0.929, 2.195)	1.246 (0.56, 2.773)	1.285 (0.563, 2.932)
HFRS (reference: no score)				
Low	1.597 (0.791, 3.226)	1.605 (0.794, 3.244)	**4.487 (1.205, 16.71)**	**4.585 (1.229, 17.12)**
Intermediate	1.645 (0.996, 2.717)	**1.767 (1.066, 2.927)**	**4.662 (1.62, 13.417)**	**4.852 (1.677, 14.04)**
High	1.298 (0.777, 2.168)	1.374 (0.82, 2.303)	2.511 (0.797, 7.913)	2.574 (0.813, 8.15)
Vaccine (reference: Oxford–Astrazeneca)				
Pfizer-BioNTECH	**3.362 (2.166, 5.216)**	**3.829 (2.452, 5.979)**	1.921 (0.727, 5.077)	2.196 (0.821, 5.873)
Observations	13,989	13,989	13,605	13,605
Events	97	97	27	27
Concordance (Standard Error)		0.669 (s.e. 0.028)		0.693 (s.e. 0.052)
Global Schoenfeld residual				
Chi-squared		8.9		7.8
Degrees of freedom		7		7
*P* value		0.26		0.35

**Table 3:** Univariable and multivariable Cox proportional hazards models with 7 and 21-day landmark times applied to the cohort and a maximum 14-day observation period. HRs and presented with 95% CIs. Results that are statistically significant at the 95% level are highlighted in bold font

	7-day landmark, 14-day observation		21-day landmark, 14-day observation	
HRs (95% CI)	Univariable	Multivariable	Univariable	Multivariable
Age	0.998 (0.97, 1.025)	1.005 (0.977, 1.034)	1.012 (0.963, 1.064)	1.014 (0.963, 1.068)
Previous positive PCR test (reference: no)				
Yes	0.549 (0.273, 1.102)	**0.435 (0.215, 0.881)**	0.808 (0.276, 2.364)	0.693 (0.233, 2.055)
Sex (reference: female)				
Male	1.526 (0.952, 2.446)	1.584 (0.969, 2.589)	1.025 (0.425, 2.471)	0.995 (0.402, 2.462)
HFRS (reference: no score)				
Low	1.047 (0.438, 2.505)	1.04 (0.434, 2.49)	2.247 (0.436, 11.58)	2.307 (0.447, 11.911)
Intermediate	1.136 (0.631, 2.045)	1.226 (0.679, 2.216)	**4.251 (1.453, 12.436)**	**4.479 (1.523, 13.169)**
High	1.027 (0.576, 1.829)	1.095 (0.612, 1.958)	2.528 (0.802, 7.967)	2.653 (0.838, 8.401)
Vaccine (reference: Oxford–Astrazeneca)				
Pfizer-BioNTECH	**3.78 (2.307, 6.194)**	**4.316 (2.616, 7.121)**	1.646 (0.563, 4.817)	1.917 (0.647, 5.682)
Observations	13,989	13,989	13,605	13,605
Events	73	73	24	24
Concordance (Standard Error)		0.684 (s.e. 0.032)		0.681 (s.e. 0.056)
Global Schoenfeld residual				
Chi-squared		6.2		14.5
Degrees of freedom		7		7
*P* value		0.51		0.04

## Discussion

Our study focussed on older adults resident in care homes. This is a particularly vulnerable sub-population that has not previously been studied in relation to infection rates for SARS-CoV-2 following vaccination, and which has suffered considerably from the most severe effects of the pandemic. Our study used a large cohort of 14,104 individuals, which is comparable to the entire case population of the ChAdOx1 nCoV-19 vaccine (N = 12,174) [4]. We were also able to include information on frailty, previous infections and vaccination received.

We found 148 (1.05%) of individuals in our cohort had a positive PCR test following vaccination. The Kaplan–Meier curve and empirical CDF suggest a susceptible period for infection of up to 42 days, with approximately 99% of infections occurring within this period. It is well known that there is a delay following immunisation for the vaccine to be effective, but this highlights the need for extended vigilance during this period for this highly vulnerable care home population. For example, extra precautions should be taken for visitations within care homes during this period.

We found a large, and statistically significant reduced risk for infection post-vaccine for individuals who had already had a SARS-CoV-2 infection. The risk was approximately halved, and those who had a prior infection may be more robust and have existing antibodies, leading to a reduced risk of subsequent infections. Increased levels of frailty, determined by the HFRS, were associated with substantially increased risk of infection post-vaccine (up to almost 5-fold increase for intermediate frailty in the 21-day landmark post-vaccination). Frailty is complex, and those living with high levels of frailty may need additional support. In particular, increased care requires additional contact with carers, and subsequently an increased risk of transmission of SARS-CoV-2 compared with those who are more independent or can be isolated. We note that the effect was not quite as large in the highest frailty group. This simply may reflect uncertainty in the risk estimates, or may be the result of more complex management or identification of risk in this group.

In the 7-day landmark analysis, there was evidence to suggest an increased risk of infection post-vaccination for those receiving the Pfizer-BioNTECH vaccine compared with the Oxford–AstraZeneca vaccine, but no statistically significant difference in the 21-day landmark analysis. This suggests the Oxford–AstraZeneca vaccine has a potentially shorter time to become effective in our cohort.

### Strengths

This analysis was possible using the existing infrastructure of the SAIL Databank, and we will continue to investigate adverse events for individuals receiving a vaccination. We were able to rapidly develop and analyse a large cohort of care home residents with the inclusion of individual level information.

### Limitations

Because of the nature of the vaccination rollout, we only had a limited follow-up time and subsequently a small number of events. At the time of analysis, we did not include information of second doses because of very small numbers. We will continue to monitor the care home population and will update our analysis with an extended follow-up time and second doses when possible. In further work, we aim to include background prevalence of COVID-19 to help account for the change in incidence over the study period. We also aim to include multi-morbidities and additional measures of frailty that do not rely on hospital data, such as the electronic frailty index [16, 17], and we will investigate additional adverse events such as mortality and hospitalisation.

## Conclusion

Our findings suggest care home residents with frailty are the most susceptible to infection post-vaccination and should be prioritised for a second dose of the SARS-CoV-2 vaccine. We also found a susceptible period of reinfection of up to 42 days, indicating extra care and precautions should be taken in this period.
